# Assessing cerebral capillary function and stalling using single capillary reporters in ultrasound localization microscopy

**DOI:** 10.1073/pnas.2509564123

**Published:** 2026-01-09

**Authors:** Stephen A. Lee, Alexis Leconte, Alice Wu, Joshua Kinugasa, Gerardo Ramos Palacios, Jonathan Porée, Abbas F. Sadikot, Andreas Linninger, Jean Provost

**Affiliations:** ^a^Department of Engineering Physics, Polytechnique Montreal, Montreal, QC H3T 1J4, Canada; ^b^Department of Biomedical Engineering, Chiba University, Chiba 263-8522, Japan; ^c^Department of Neurology and Neurosurgery, McGill University, Montreal, QC H3A 2B4, Canada; ^d^Montreal Neurological Institute and Hospital, Montreal, QC H3A 2B4, Canada; ^e^William Cone Laboratory for Neurosurgery Research, Montreal, QC H3A 2B4, Canada; ^f^Department of Biomedical Engineering, University of Illinois, Chicago, IL 60607; ^g^Montreal Heart Institute, Montreal, QC H1T 1C8, Canada

**Keywords:** ultrasound localization microscopy, microvasculature, capillary stalling, neuroimaging

## Abstract

While transcranial neuroimaging of individual capillary function holds transformative potential for diagnostics, it has proven difficult to achieve. Superresolution ultrasound, while capable of achieving micron-scale resolution, relies on the accumulation of multiple microbubble events, a method inherently limited by the exceedingly low probability of observing such events within capillaries. We present single capillary reporters (SCaRe), a paradigm-shifting approach that utilizes the complete flow trajectory information extracted from individual microbubbles to directly image single capillaries. This method allows for transcranial reconstruction and functional assessment of deep capillary networks in the entire brain. We employed computational simulations and pathological neuroinflammation models to quantify and validate metrics such as capillary transit-time and capillary stalling. Importantly, we demonstrated SCaRe’s ability to resolve immune responses to injury at the single capillary level, markedly broadening research avenues for exploring microvascular dysfunction across diverse neurological conditions.

## Significance

Growing evidence indicates that the brain’s microvascular system plays a key role in neurological diseases and aging. This is especially true for capillaries, the smallest blood vessels, that interact directly with neurons. However, imaging and measuring the function of these tiny vessels through the skull remains extremely challenging. Here, we introduce single capillary reporters (SCaRe), statistically derived biomarkers that track the movement of individual microbubbles to directly image and assess single capillaries in ultrasound localization microscopy. SCaRe surpasses the spatial and temporal limitations of current techniques, enabling the extraction of markers that reflect capillary function in both healthy and diseased brains. These biomarkers reveal immune-related responses to brain injury with single capillary precision.

Neuronal health is intrinsically linked to the function of the microvasculature (1). Emerging evidence suggests that disruptions in microvascular function are associated with the onset, maintenance, and progression of neurodegenerative conditions (2). Notably, at the capillary level, where oxygen exchange occurs, red blood cell (RBC) velocity is at its lowest. This is of particular importance since anomalies in capillary flow dynamics, such as turbulent flow, endothelial damage (3), or intermittent RBC stalling (4), have been correlated with cognitive decline and worsening neurodegeneration (5). Furthermore, excessive capillary stalling at the single capillary level can trigger downstream effects, disrupting the microvasculature and their associated neural networks (6). Since each capillary supports a cluster of neurons (7), there is a critical need for advanced in vivo imaging techniques that can precisely assess single capillary function and stalling throughout the brain. While MRI, micro-CT, and two-photon imaging can assess either overall vascular structure or cumulative measurement of transit-time (8), they are limited either by depth or resolution; no imaging method currently enables high-resolution transcranial assessment of individual capillary transit-times or stalling.

Transcranial ultrasound imaging techniques, such as Doppler-based methods, have been developed to reconstruct the brain vasculature (9). Additionally, high-frame-rate functional ultrasound (fUS) can detect stimulus-evoked responses by measuring changes in cerebral blood volume as a proxy for neuronal activity (10, 11). However, these approaches remain limited by acoustic diffraction and are therefore unable to resolve individual capillaries. Ultrasound localization microscopy (ULM) offers a promising avenue for whole-brain microvascular imaging, uniquely enabling noninvasive, deep-tissue visualization of microvascular morphology (12, 13). By tracking circulating microbubbles, ULM reconstructs superresolved vessel structures and blood flow velocities at the micrometer level (12, 14). However, this capability hinges on the observation of multiple trackable microbubbles traversing the same vessel. Moreover, structural imaging and flow velocity mapping offered by traditional ULM offer limited functional information, presenting a gap in the clinical translatability of ULM. Dynamic ULM (dULM) captures functional information like pulsatility through cardiac cycle synchronization (15, 16). Functional ULM (fULM), a superresolved form of fUS, allows additional imaging of different neurovascular responses, including to somatosensory stimuli (17). Notably, the detection of renal glomeruli through microbubble rotation (18, 19) illustrates the versatility of ULM for novel functional imaging. Yet, these ULM variants assess the function based on larger vasculature, still leaving in question the utility ULM in single capillary imaging.

Microvascular mapping in ultrasound is premised on the ability to remove stationary clutter, such as the skin, skull, and brain tissue, from moving, incoherent, signals like the blood or contrast agents where microbubbles assume blood flow speed (20). Conventional clutter filtering approaches typically involve finite impulse response (FIR), or infinite impulse response (IIR) filters applied along the slow-time dimension (21, 22), or nonlinear techniques that leverage the distinct scattering properties of microbubbles to suppress tissue signals (23). However, when fast-moving tissue overlaps with slow-flowing blood, these filtering methods struggle to cleanly separate the signals.

Recently, singular value decomposition (SVD)-based clutter filtering has been introduced to exploit the redundancy in ultrafast ultrasound data, enabling the separation of blood and tissue signals based on their spatiotemporal coherence (24). The signal can be decomposed into singular components, where tissue typically dominates the highest singular values. The subsequent removal of these largest eigenvalues and reformulation of the dataset results in primarily moving blood signal. However, key challenges remain—specifically, how to optimally choose ensemble length and cutoff thresholds to recover slower flows. As evidenced by Song et al. (25), block-wise SVD results in spatially varying singular value cutoffs required for local separation of tissue and blood.

This challenge is particularly acute in capillary imaging, where flow is both slow and spatially sparse. Capillary flow is difficult to distinguish from tissue motion, especially during transcranial imaging where signal degradation from skull-induced aberration and attenuation further complicates separation. The introduction of contrast agents to the microvasculature used to map the microvasculature with ULM can alleviate some of these problems. However, ULM still relies on differentiating moving microbubbles from static tissue using clutter filtering techniques and are unable to resolve the slow flows (<5 mm/s) necessary for direct transcranial imaging of single capillary tracks using ULM or fULM (26), due to interference from slow moving tissue clutter. Even if slow flow recovery was technically feasible, it would still be insufficient for functionally resolving individual capillaries due to the inherent sparsity and infrequent occurrence of microbubble events. It is estimated that it takes tens of minutes to perfuse the capillary bed fully with microbubbles (27, 28). Specifically, due to the concentration of microbubbles, the ultrasound acquisition parameters, and the physiology of the animal, complete perfusion can take at least 10 min to hundreds of minutes. Moreover, the vast number of flow paths makes repeated microbubble traversals through the same capillary during a typical ULM acquisition rare. These limitations highlight the need for new strategies—both in acquisition and analysis—to capture and functionally assess capillary-level dynamics.

Here, we depart from conventional superresolution imaging by posing a fundamental question: “Can a single microbubble trajectory reveal its location within the vascular tree?” To address this, we introduce single capillary reporters (SCaRe)—a framework for transcranial reconstruction of whole-brain capillary networks. SCaRe integrates long-ensemble spatiotemporal filtering of continuous ultrasound data with hidden Markov model-based velocity state estimation to recover the whole vascular path of a microbubble through the brain. Within this framework, we define “dwell time” as the duration a microbubble takes to traverse a reconstructed capillary path—its distribution reflecting capillary transit time heterogeneity—and “stall time” as a transient interruption in capillary flow that may indicate impaired or pathological vascular function. We 1) validate SCaRe using in silico computational models and hemodynamic brain models, 2) demonstrate its utility in a neuroinflammation model for assessing in vivo single capillary function, and 3) confirm associations between capillary stalling and microglial activation through histochemistry.

## Results

### Unique Capillary Dynamics Are Highlighted Using In Silico Realistic Mouse Microvascular Networks of Microbubble Flow.

**Fig. 1.**
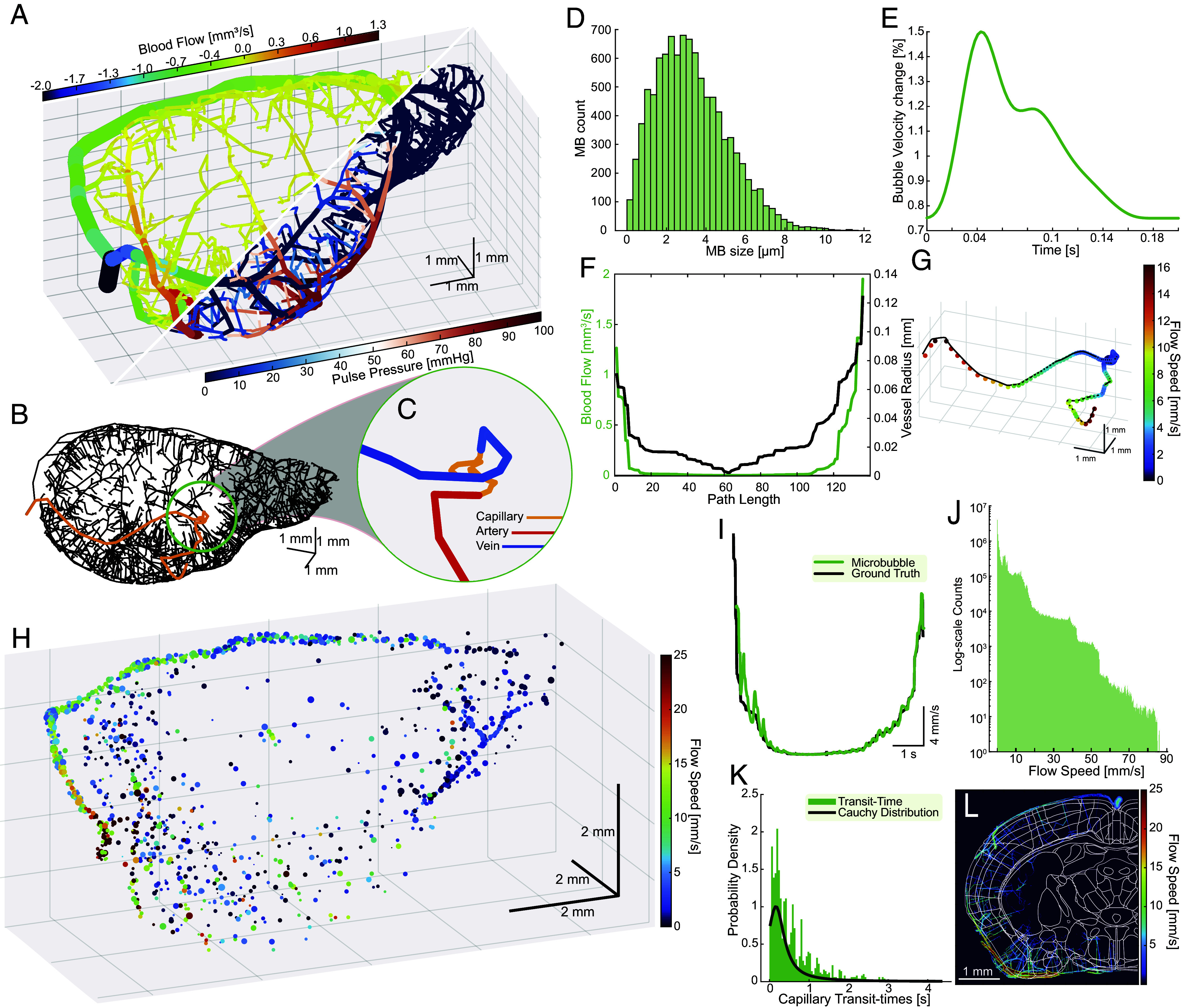
Realistic microvascular phantoms demonstrate unique microbubble behaviors in capillary vessels. (*A*) Mouse brain microvascular network with fully connected capillary networks. Top axis indicates vessel diameter and direction of blood flow (sign) (*Top*), bottom axis shows pulse pressure. (*B*) Skeletonized vascular network with a single full vascular path identified. (*C*) Zoomed color-coded image of a single vascular path. (*D*) Simulated Definity microbubble distribution from which simulated results are sampled. (*E*) Sample pulsatile waveform calculated using pulse decomposition analysis. (*F*) Sample blood flow and microvessel radius of the path depicted in *B* and *C* used to forward propagate simulated microbubbles. (*G*) Resultant microbubble trajectory along path depicted in *B* and *C* using initial conditions in *D*–*F*. (*H*) Representative fully populated microbubble dataset through the synthetic mouse brain (Movie S1). (*I*) Distinct U-shaped velocity profile of the path depicted in *B* and *C*, and *G*. (*J*) Log histogram of sampled microbubble velocities throughout the whole dataset in *H*. (*K*) Sampled capillary transit-times, measured as time required for a microbubble to traverse a capillary network, fitted with a Cauchy distribution. (*L*) Ground truth microbubble paths with the Allen Institute mouse brain atlas (Plate 61) overlaid on top, showing regional differences in microbubble flow speed.

Among the various computational models of microvascular flow in the rodent brain, we used synthetic networks based on state-of-the-art optical neuroimaging data to demonstrate unique and trackable microbubble behaviors in capillaries (Fig. 1*A*). These networks emulate complete and balanced circulation, encompassing fully connected capillary closures and topology from arteries to veins (for a full description of the image-based synthesis and hemodynamic simulation of full mouse brains see refs. 29 and 30). Our hemodynamic simulations can predict oscillatory blood pressure and flow rates at nodes and edges of directed graphs using a graph theoretical approach developed for large scale vascular anatomical networks (31, 32). By constructing a dataset of microbubbles flowing through the microvasculature, we can retrieve all feasible paths from inlets (arteries) to outlets (veins), traversing a single capillary (Fig. 1 *B* and *C*). We quantified movement, microbubble velocity, and transit time behavior throughout the capillary network, given blood flow, vessel radius, and pulse pressure as computed with methods described in refs. 33 and 34.

We generated synthetic microbubble ultrasound data, via sequential Monte Carlo simulations, randomly sampling specific paths (Fig. 1 *B* and *C*) given initial conditions (Fig. 1 *D*–*F*). Using a mouse brain hemisphere, this approach yielded a total of 291,372 potential paths (876,161 nodes; 1,167,529 edges) for microbubble flow (Movie S1). For each path, we sampled from a microbubble distribution, modeled as a polydisperse bolus of Definity microbubbles (<12 μm), comparable to the average diameter of a RBC (~7.5 μm), to mimic an in vivo bolus injection (35) (Fig. 1*D*). Here, simulation boundary conditions were imposed such that a larger microbubble may not traverse a capillary with a smaller diameter, thus simulated microbubble paths may be biased toward larger capillaries. Subsequently, we calculated the associated pulsatile waveform via pulse decomposition analysis (36), applying a 75% variation from peak systole to peak diastole in blood velocity for all vessels (Fig. 1*E*). Radially distributed blood flow velocities for each vessel segment and instance in time were computed from the graph-based volumetric blood flow rates under parabolic profile assumptions (32). Thus, instantaneous flow speed and vessel diameters were retrieved (Fig. 1*F*) and used to calculate bubble velocity and radial distance from the centerline along the vessel path (37). Finally, we forward propagated the selected microbubbles from inlet to outlet based on the initial conditions and traveling pulse wave velocity (38, 39). Simulation parameters can be found in *SI Appendix*, Table S1.

Fig. 1*H* illustrates a fully populated mouse brain hemisphere microvascular network following seeding with randomly sampled microbubble paths (Movie S1). Here, the key insight was that distinct U-shaped velocity patterns (40) can be used to distinguish a single microbubble from all other trajectories that traverse the arteriovenous system, and where low velocities (<5 mm/s) indicate travel through the capillary mesh (Fig. 1*I*). Given such a trajectory, we can relay where the capillary is and quantify how long the microbubble “dwells” in the mesh as an indicator of capillary transit-time. In this dataset, microbubble velocities range from 0 to 85 mm/s (Fig. 1*J*), where capillary vessels had velocities of less than 2 mm/s. Furthermore, we can estimate the capillary transit-time distribution within the whole vascular network, measured as the time it takes for a bubble to move from the start to the end of a capillary (areas of velocity < 2 mm/s) (Fig. 1*K*). We find that the estimated capillary transit-time appears to conform to a Cauchy Distribution, chosen to best model long transit times (41). Furthermore, we can measure regional transit-time (Fig. 1*L*) and start identifying spatiotemporal patterns. Crucially, we see that transit-times can be as long as 4 s, indicating that current ULM techniques (~0.5 to 1 s continuous acquisitions with pauses in between) are under sampling capillary temporal dynamics.

### Capillary Transit-Time Necessitates Long and Continuous High-Frame-Rate Acquisition.

**Fig. 2.**
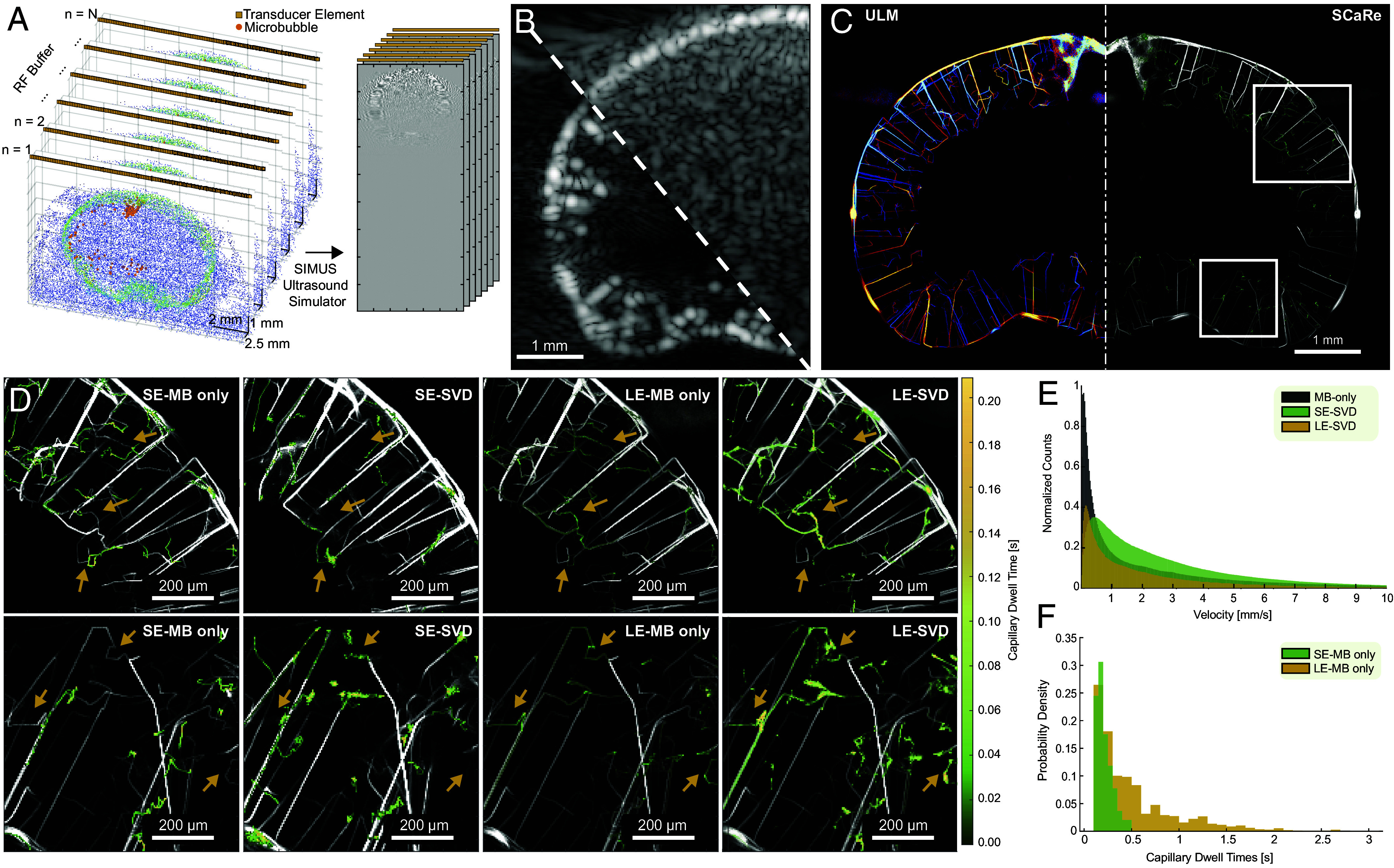
SVD clutter filtering radically changes ULM capillary recovery in in silico brains. (*A*) Ultrasound data generation pipeline from scatterer distribution [microbubbles (orange) and tissue scatterers are assigned intensity according to microCT] & probe orientation to RF data simulation. (*B*) Representative simulated beamformed data, with and without clutter. (*C*) Representative superresolution ULM-SCaRe maps (red/blue indicates downward and upward flow, green indicates capillary dwell time) of accumulated tracks over 350 buffers (175 s) using long ensemble microbubble only (LE-MB). (*D*) Zoomed images of the ROIs found in *C*. where the top row corresponds to the top ROI, and the bottom row to the bottom ROI. From left to right: Short Ensemble microbubble only (SE-MB) only, SE-SVD (20/1,000 eigenvalue high-pass cutoff), LE-MB only, long ensemble SVD (LE-SVD) (20/6,000 eigenvalue cutoff). (*E*) Probability density histograms of velocity changes between tracked microbubble-only velocities and SVD filtered velocities in SE conditions. (*F*) Probability density histogram of tracked capillary transit-time for SE vs. LE.

Our results indicate that a factor preventing capillary imaging is the time required to track the complete microbubble behavior, necessitating continuous and long acquisition times. In fact, it is estimated that complete reconstruction of capillary vessels using ULM would take several minutes (27). Thus, we hypothesize that the low velocities found within capillaries may be filtered out when using clutter filtering techniques like the SVD due to signal overlap. To investigate the impact of SVD clutter filtering on slow-moving microbubbles, we simulated realistic ultrasound data with added skull clutter on the previously simulated MB dataset. Leveraging an open-source dataset of mouse micro-CT scans (42), we generated cluttered skull signals with injected movement to mimic SVD clutter filtering behaviors and artifacts (43) (Fig. 2 *A* and *B* and Movie S2). From here, we trained a Hidden Markov Model to estimate two states: high velocity and low velocity and delineate patterns of the estimated states as a capillary. We accumulate tracks to map directional density (Fig. 2 *C*, *Left*) and SCaRe overlaid on the vasculature (Fig. 2 *C*, *Right*).

We investigate four conditions [short ensemble microbubble (SE-MB) only, short ensemble SVD (SE-SVD) filtered, long ensemble microbubble LE-MB only, and LE-SVD)], where microbubble only indicates no tissue clutter. Specifically, we investigate removing 20 eigenvalues out of an ensemble size of 1,000 (1 s), for short ensemble (20/1,000), and 6,000 (6 s), for long ensemble (20/6,000). By tracking the microbubbles over time, we see that short ensemble SVD (SE-SVD) filtering significantly hinders the recovery of slow-flowing tracks associated with capillaries. Within the zoomed ROIs (Fig. 2*D*) we see that short ensembles with gaps in acquisition time cannot adequately localize capillary behaving microbubbles because the acquisition times (0.5 s) are too short to sample the whole trajectory. This results in insufficient time to sample the whole U-shaped capillary path and incorrect HMM state estimation. Adding the additional SVD layer results in the same capillary behavior (missing or incomplete tracks shown via arrows). Quantifying DICE score, Jaccard Index, Sensitivity, and Specificity indicates that higher SVD high-pass cutoffs decrease precision of localizations (*SI Appendix*, Fig. S1). Alternatively, if we increase the ensemble size (LE-MB only, LE-SVD), we can better recover the synthetic vasculature, as well as better capillary tracking. We can effectively recover the expected ground-truth capillary tracks in both conditions. We see however, due to SVD filtering, our ability to estimate capillaries, although effective, have lost some detail and resolution.

Furthermore, Fig. 2*E* shows that SVD filtering alters the possible recoverable velocities with fewer slow flow velocities. Compared to SE-MB only, SE-SVD has shifted the velocity distribution to the right, indicating less recovered slow flow velocity tracks. However, with LE-SVD the distribution maintains the same shape as with the MB-only condition. With regard to capillary transit time, Fig. 2*F* illustrates that SCaRe is more effective with tracking over long ensembles with a 500% increase in max capillary time (*SI Appendix*, Fig. S2).

### LE-SVD Recovers Slow Flow Microbubbles In Vivo.

**Fig. 3.**
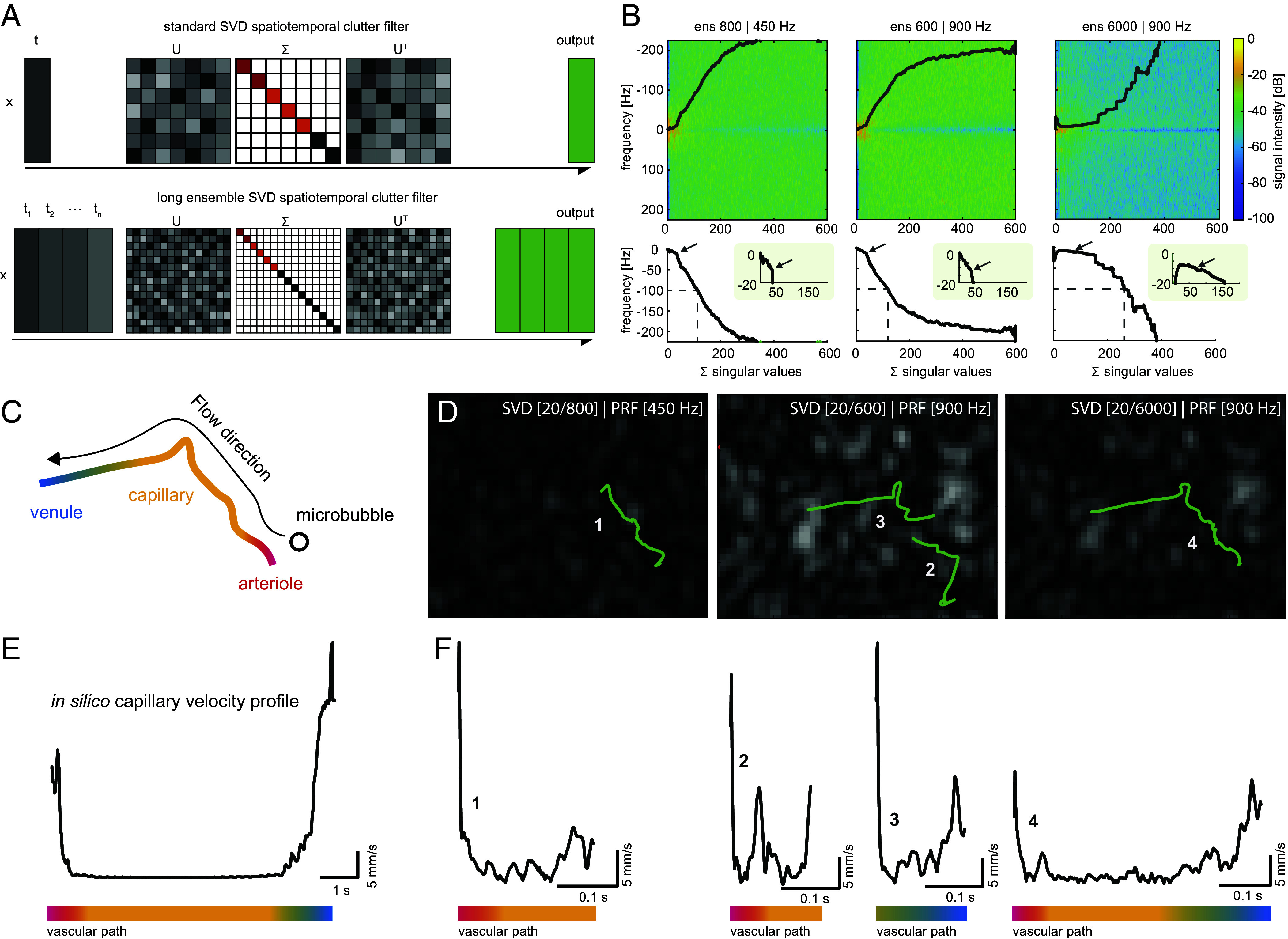
SCaRe can recover the whole arterio-venous transit path. (*A*) Illustration of SVD clutter filtering and LE-SVD methodology. (*B*) Representative power spectrum of three different conditions (800 SE-450 Hz, 600 SE-900 Hz, 6,000 LE-900 Hz) and their respective mean power spectrum as a function of sorted singular values. Zoom panels are centered around the first inflection point of spectral density. (*C*) Illustration of an example in vivo microbubble flowing through the vascular path from an arteriole through a capillary to a venule. (*D*) Representative tracking results with identical parameters over 3 SVD clutter filtering conditions for a single vascular path. The number corresponds to velocity profiles found in *F*. (*E*) Velocity behavior of a microbubble through a whole vascular path in simulated results for comparison to *F*. (*F*) Velocity profiles for in vivo tracks of a single microbubble over 3 SVD conditions corresponding to numbers indicated in *D*. The colorbar in *E* and *F* represents the vascular anatomical path in *C*.

Traditional SVD clutter filtering hinders our ability to recover the full U-shaped velocity profiles, creating a gap where stationary microbubbles are eliminated, making clear the need for the acquisition of continuous data to increase ensemble sizes to maximize the likelihood of capturing full capillaries behaviors. Thus, ULM sequences recorded noncontinuously for less than 4 s may be insufficient to adequately sample capillary transit-times. To test this, we apply LE-SVD in vivo to retain the full velocity spectrum necessary for recovering U-shaped microbubble velocity behaviors (Fig. 3*A*).

Next, we investigated the effects of ensemble size on in vivo brain imaging in wild-type C57BL/6 mice. Mice were tail vein catheterized and injected with a 1:20 dilution of Definity microbubbles (4 μL/kg body weight) in physiological saline, delivering a maximum of approximately 4.8 × 10^7^ microbubbles per kilogram. Then, we compare, in vivo, two conditions (450 & 900 Hz frame rates) of SE-SVD to LE-SVD at 900 Hz compounded frame rates. To quantify how the subspaces change, we calculated the dominant temporal frequency of each singular vector and plotted these as a function of singular value index (Fig. 3*B*). Following prior work (24, 44), singular vectors with dominant frequencies below ~100 Hz correspond to stationary tissue motion (brain tissue & skull); thus, the reported singular values (SE-SVD:112, 119, LE-SVD: 262) represent the number of singular vectors that comprise dominant frequencies below this 100 Hz tissue threshold. We also identified the inflection point of each curve, which is theorized to mark the transition between tissue-dominated and blood-dominated subspaces. Here, this occurred at singular vectors 36 and 37 for the SE-SVD conditions and at 76 for LE-SVD. In both respects, LE-SVD doubles the corresponding number of singular values to SE-SVD conditions and because LE-SVD provides longer ensemble sizes, it facilitates a clearer separation of tissue clutter from slow blood-flow signals. Although these inflection points served as guides for selecting SVD cutoffs, in LE-SVD we found that removing only the first 20 singular values was sufficient to suppress tissue clutter while preserving slow-flow microbubble signals. Experimentally, we therefore set the eigenvalue cutoff to the minimum value required to eliminate significant tissue contamination.

Tracking results for three conditions of the same microbubble and vessel in the mouse brain are illustrated in Fig. 3 *C* and *D* overlaid on a single B-mode still after SVD filtering. While low frame rates may recover slow moving microbubbles by increasing their displacement in time, they inevitably alias fast-moving flow into and out of the capillary, losing the entire trajectory in the process. Conversely, high frame rates capture high velocities but miss the MB at low velocities due to insufficient sampling in the SVD, hindering full vessel tracking. In contrast, LE-SVD processed frames produce the entire estimated track, including entry, traverse, and exit from the capillary track (Movie S3). This trend is also seen in velocity profiles in silico (Fig. 3*E*) compared to associated profiles in Fig. 3*F*.

### SCaRe Biomarkers Reveal Capillary Dwell Time Throughout the Whole Brain In Vivo.

**Fig. 4.**
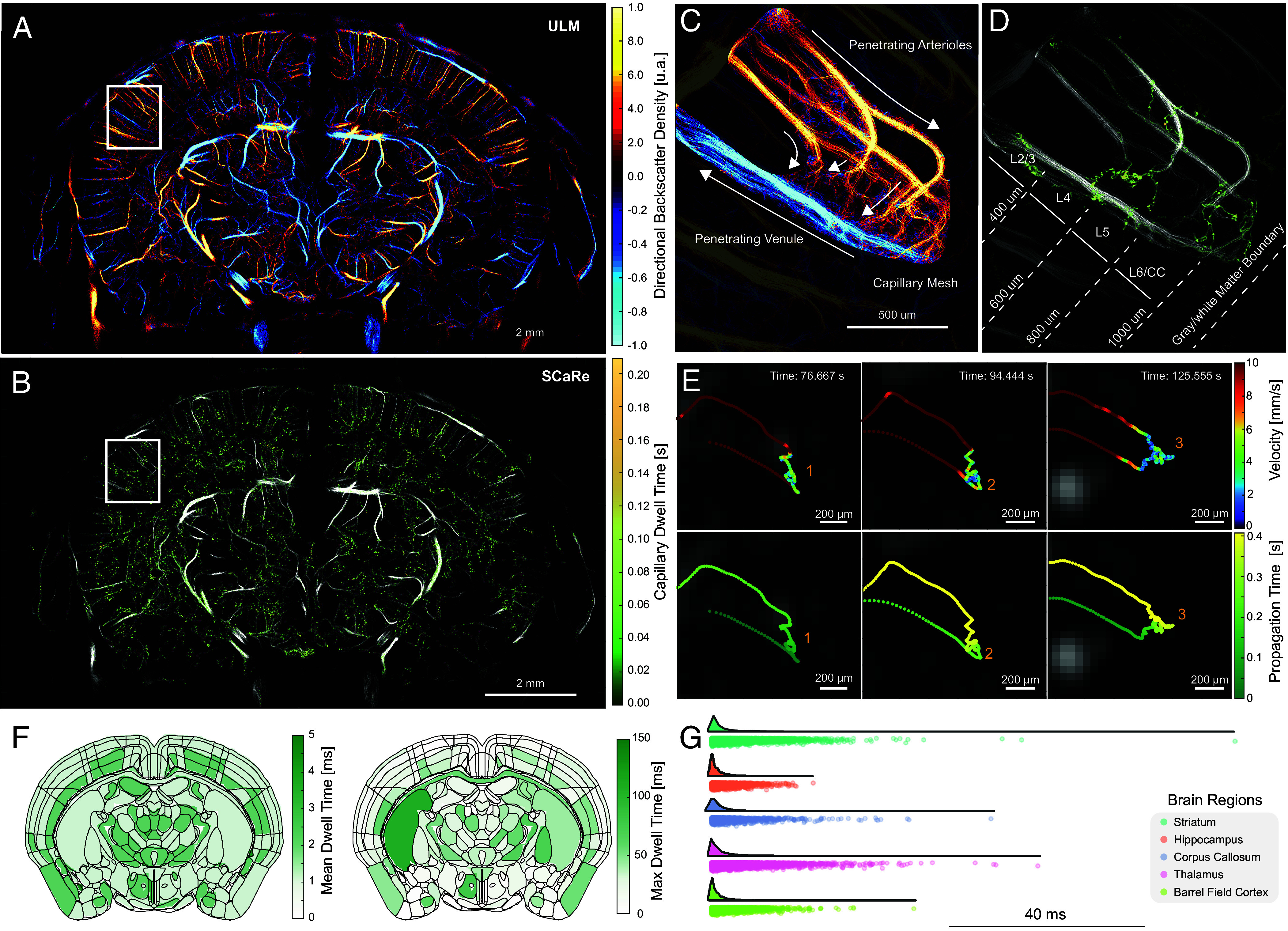
SCaRe can recover the whole arterio-venous transit path transcranially in vivo. (*A*) Representative in vivo ULM maps of a wild-type mouse brain using LE-SVD and track pairing. (*B*) Representative whole brain SCaRe map of measured capillary tracks with color bar representing the pixel-wise capillary dwell time (time a microbubble rests in a pixel) at Bregma = −1.5 mm. (*C*) Zoom panel in *A* of a completed vascular path with descent in the penetrating arterioles (red), the ascent out of the penetrating venule (blue), and the capillary mesh in between. (*D*) Zoom panel in *B* illustrating tracked capillary networks in neuronal layers 5 and 6. (*E*) Tracks from three different capillary paths through the different arterioles to the same venule separated by microbubble velocity and time of travel through the vascular network. (*F*) Parcellated brain regions (plate 68 Allen Brain Atlas) demonstrating mean (*Left*) and maximum (*Right*) dwell time across all brain regions at the baseline in wild-type mice. (*G*) raincloud plots show the distribution of the capillary dwell times for the Striatum, Hippocampus, Corpus Callosum, Thalamus, and the Barrel Field Cortex.

Here, we performed comprehensive whole-brain SCaRe mapping in vivo using continuous, gapless data acquisition. Gapless acquisition was achieved by ensuring that the time required to write a dataset to the disk (acquired at a 1,000 Hz compounded frame rate) matched the time needed to acquire the next dataset in a circular buffered fashion. Because the data were acquired without temporal discontinuities, SCaRe processing can be conducted via post hoc stitching for dataset equivalent to a total of 6-s. Fig. 4*A* illustrates a track density map using backscattering amplitude (45) for display and Fig. 4*B* shows the corresponding SCaRe map with distinct single capillary tracks discernible in both cortical and subcortical regions of the brain using a 15 MHz array. *SI Appendix*, Fig. S3 illustrates SCaRe composed using a 10 and 15 MHz linear transducer. Zoomed panels in Fig. 4*C* isolate single vascular paths composed of a penetrating arteriole connected to penetrating venules. Remarkably, this map illustrates a composite of MB paths originating from several arterioles (red; descent) leading to a single venule (blue; ascent).

Furthermore, Fig. 4*D* showcases the ability to segregate measured capillary transit time by neuronal layers. This capability is particularly pertinent given previous studies indicating layer-specific differences in capillary function, notably in age-related white matter loss (46). Individual SCaRes can be analyzed to discern their travel path, velocity behaviors, and estimated capillary transit time via dwell time (time spent in a pixel), as depicted in Fig. 4*D*. In accordance with our simulations, capillaries identified using SCaRe exhibit high-velocity entry, followed by low-velocity capillary transit, and culminating in high-velocity uptake into penetrating venules. Notably, Fig. 4*E* illustrates velocity and propagation time for these specific capillaries, tracked over an entire minute (Movie S4). Here, we can identify exactly when the microbubble enters a region of low velocity (<2 mm/s) with differences in the microbubble travel path within the capillary mesh. The propagation time shows that these microbubbles enter from the same arteriole and exit from the same venule with varying transit-times (Movie S5). Last, the number of localized arteriole-capillary-venous units can be changed based on the concentration of the microbubbles, the imaging frequency, as well as the type of injection (bolus vs. continuous infusion). Our simulations suggest that per dataset, we can acquire 10.8 ± 1.5 capillaries/s (3,235 ± 450 total). This is consistent with our 5-min continuous infusion in vivo measurements of 7.4 ± 2.2 capillaries/s (2,220 ± 660 total) at a 15.625 MHz frequency vs. 5.9 ± 1.9 capillaries/s (1,770 ± 570 total) at a lower frequency (10 MHz) for a bolus injection and scanning for the same duration. Estimates of capillaries were performed for n = 3 wildtype C57BL6 mice.

Baseline dwell times were quantified across brain regions (Fig. 4 *A* and *B*) using plate 68 of the Allen Brain Atlas (Fig. 4*F*). We observed that deeper cortical layers, as well as several subcortical regions within the thalamus, exhibited elevated mean dwell times. By contrast, analysis of maximum dwell times recorded during the 5-min scan revealed that subcortical regions overall displayed slower transit dynamics. To further examine regional variability, we quantified baseline dwell times in the striatum, hippocampus, corpus callosum, thalamus, and barrel field cortex (Fig. 4*G*). White matter (corpus callosum) and subcortical regions (striatum and thalamus) demonstrated longer dwell times than the barrel field cortex. Interestingly, the hippocampus exhibited relatively shorter transit times, suggesting potential differences in vascular organization or flow regulation between gray and white matter regions.

### SCaRe Capillary Transit Time and Stalling Biomarkers Are Associated with Microglia Activation in Neuroinflammation.

**Fig. 5.**
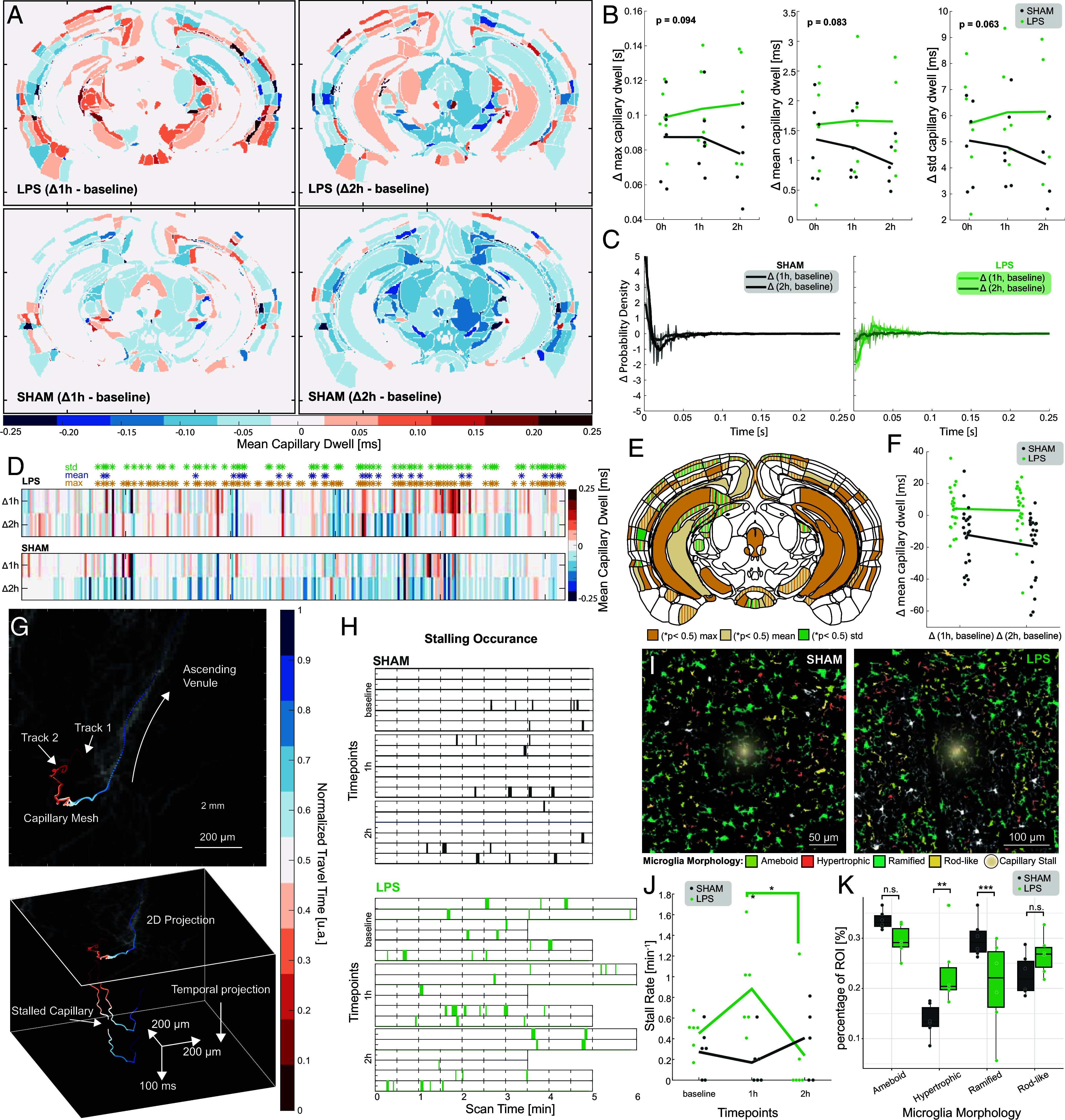
SCaRe can quantify capillary dwell time and stalling throughout the whole brain during neuroinflammation. (*A*) Representative baseline-subtracted difference maps of mean capillary dwell time at timepoints 1 and 2 h after LPS-challenge. (*B*) Quantification of dwell time changes in max, mean, and SD over three timepoints. (*C*) Mean difference between timepoints and baseline probability density functions of recorded capillary dwell times for SHAM (*Left*) and LPS (*Right*) injection. (*D*) Heat-map of brain region analysis of capillary dwell times on the Allen Mouse Brain Atlas (Plate 83). Significant ROIs for changes in max, mean, and SD are denoted by an asterisk (*P < 0.05, paired *t* test with false discovery rate correction). (*E*) Significant brain areas in *D* with overlapping areas illustrated in stripes. (*F*) Mean baseline-subtracted capillary dwell times for each ROI in *E*. (*G*) Visual representation of capillary stalling in 2D (*Top*) and 3D w.r.t. time (*Bottom*) in Layer 6 of the Visual Cortex. (*H*) Stalling occurrence at each timepoint over the whole scan time duration. (*I*) Representative histology images of microglia stained with Iba1 antibodies taken at stalling areas (hotspot) for SHAM and LPS mice. Microglia morphology using cluster analysis is overlaid on top. (*J*) Capillary stalling rate over 3 timepoints (* *P* < 0.05, 2-way ANOVA multiple comparisons). (*K*) percentage of microglia distributions within stalling ROIs (**P < 0.01, ***P < 0.001, Generalized Linear Mixed Models with Bonferroni Correction).

Last, we asked whether SCaRe biomarkers could detect sensitive changes in capillary function in normal physiology and under duress at the single capillary level throughout the whole brain. To induce neuroinflammation, we used an intraperitoneal (IP) lipopolysaccharide (LPS) challenge to increase neutrophil circulation and subsequent capillary stalling (47, 48) in n = 6 mice compared with n = 6 PBS-vehicle SHAM mice. We localized capillary dynamics over three time points, baseline, 1 h-post, and 2 h-post (Movie S6). Here, we quantified the length of time that a microbubble spends in a capillary, coregistered with the Allen Brain Atlas (plate 83). Fig. 5*A* demonstrates brain-wide changes in the average capillary dwell time at 1 and 2 h post LPS/SHAM injection from baseline recordings. We see an overall global change in LPS-challenge mice in contrast to an overall decrease in capillary dwell time in PBS-injected mice. The decrease in the SHAM is expected since anesthesia via isoflurane can induce blood flow increases within minutes (49) while capillary transit-times are expected to increase via LPS-induced neuroinflammation (50).

If we quantify global changes throughout the brain, we see that neuroinflammation shows a monotonic increase in maximum, mean, and SD metrics for LPS in contrast to a greater decrease in SHAM mice (Fig. 5*B*). A two-way repeated measures ANOVA indicates that changes in global dwell times over the three time points are not significantly different between groups (*P* = 0.094 max; *P* = 0.083 mean; *P* = 0.063 std; Greenhouse–Geisser adjustment). We can compare changes to capillary transit-time distributions within subjects by calculating baseline-subtracted probability density functions for each animal. The average changes are illustrated in Fig. 5*C* where we identify notable changes between the two groups. In SHAM mice, we observe a continuous shift in the distribution toward the left with increased fast transits and decreased slow transits. However, LPS-challenge instead shifts the distribution rightward, increasing the occurrence of slower transit-times while decreasing the probability of faster transit-times. This effect is greater at the 1 h timepoint than the 2 h timepoint.

Systemic neuroinflammation is expected to change the neurovascular unit heterogeneously throughout the brain (51–53); we expect changes in capillary transit times to follow similar trends. If we perform a regional analysis of the changes in each brain region, we can identify ROIs that experience significant changes. Using a two-way paired *t* test with corrections for false discovery rates, the heatmap of mean dwell time changes in Fig. 5*D* denotes brain regions with significant changes with an asterisk. These areas are illustrated on the atlas ROI map in Fig. 5*E* with each color signifying either changes in max, mean, and/or SD. Indeed, we see widespread, almost symmetrical changes, in both cortical and subcortical structures. Moreover, we see hippocampal subregions such as CA1 and CA3 have significant neurovascular responses to neuroinflammation. Last, if we plot baseline-subtracted mean dwell times for these regions, we find brain regions during neuroinflammation are elevated in comparison to PBS-SHAM regions that show a large decrease over time (Fig. 5*F*).

Due to the significant impact that capillary stalls can have on neuroinflammation, stroke, and neurodegeneration, we further asked whether SCaRe can be used to measure rare but important occurrences of stalling deep in the brain. Our HMM configuration can identify microbubble high–low (arteriole to capillary) and low–high (capillary to venule) state changes which we can pair if the capillary segments occur within 10 microns of each other. We can temporally project these tracks in 3D to visualize individual capillary stalls (Fig. 5*G*) and depict their occurrence over the minute long scans (Fig. 5*H*). Furthermore, we elucidate an association with neuroinflammation-activated microglia to capillary stalling by segmenting ROIs in Iba1 immunohistology slices at stalling vessel sites in LPS and SHAM mice (*SI Appendix*, Fig. S4) and performing morphological cluster analysis (54) of microglia states (Fig. 5*I*). Here, the hotspot was determined by convolving a Gaussian with the average location of the stall in x and z. As expected, we observe that both SHAM and LPS animals experience natural capillary stalling. Quantifying the stalling rate over the whole scan time at the 3 different timepoints, we see marked increase in LPS-challenge animals at 1 h with a decrease at 2 h (Fig. 5*J*). However, in SHAM animals, there are no large changes across all timepoints. A two-way ANOVA with tests for multiple comparisons (*SI Appendix*, Tables S2 and S3) indicates that the interaction between LPS-challenge and timepoint is statistically significant (*P* = 0.019) with a significant difference (*P* = 0.022) between the two groups at the 1 h timepoint. Importantly, analysis of the microglia morphology surrounding capillary stalls show changes in the proportions of activated microglia (Fig. 5*K*). We observe statistical significance between LPS and SHAM microglia morphology (*SI Appendix*, Table S4) with no changes in the proportion of active amoeboid and rod-like microglia. However, we see statistically significant decreased proportions of ramified (surveillant, homeostatic) and increased proportions of hypertrophic microglia that play active roles in neuroinflammation (*SI Appendix*, Table S5)

## Discussion

Noninvasive assessment of high spatiotemporal single capillary function deep within the brain has remained a significant challenge due to depth–resolution trade-offs and transcranial imaging limitations. SCaRe presents an alternative approach facilitated by continuous high-frame-rate ULM. These statistically derived markers reconstruct capillaries from a single microbubble flow pattern, enabling comprehensive quantification of deep capillary dynamics like dwell and stall time in the entire brain, transcranially, in both physiological and pathological states.

Furthermore, our technique provides a foundation for direct transcranial, single microbubble quantification of the capillary architecture. Recently, thin sound-sheet RCA nonlinear techniques have been shown to recover slow flow imaging of contrast agents through cranial windows (55). However, this requires further signal processing, nonlinear modulation, and hardware that may not have sufficient transcranial sensitivity or currently exist in the clinic; identifying slow flowing microbubbles is only a prerequisite—various factors exist in which a microbubble may encounter slow flow in the brain, including placement at the large vessel edges or pathological changes. Since the probability of imaging two or more distinct microbubbles within the same capillary is vanishingly small (27), a paradigm-change in how we classify and define vessels based on single microbubble behavior patterns is warranted allowing us to assess higher-level information on the function or health of single capillaries, such as the capillary transit time or stalling. At its core, SCaRe relies on the extraction of U-shaped velocity profiles, depicting the high-velocity descent through arterioles, slow perfusion through capillaries, and high-velocity uptake into venules. Provided that normal capillary blood flow percolates at a rate of 1 mm/s (56), there are precapillary segments that traverse at upward of 8 mm/s and thoroughfare channels may be preferentially captured due to higher flow rates. Thus, with the addition of brain pulsatility and movement artifacts, an upper-bound cutoff of 5 mm/s was chosen for in vivo characterization. While reminiscent of sensing-ULM (sULM) (18, 19) used to identify glomeruli in the kidney based on circling microbubbles, our methodology differs significantly due to the distinct behavior of brain capillaries and challenges in transcranial imaging. Moreover, because we classify capillary behaviors in SCaRe, we can attain higher confidence in measuring true capillary function.

Our results show that SCaRe can quantify capillary patterns in both cortical and subcortical regions and link these patterns to microglia morphological changes during neuroinflammation. Interestingly, we observed a nuanced response: capillary transit time positively correlated with neuroinflammation, whereas capillary stall rate initially increased but subsequently decreased. The presence of upregulated hypertrophic and downregulated stratified microglia at 2 h, without an increase in ameboid, or fully activated, morphology, suggests that the reduction in capillary stalling might be attributed to the rapid engagement of these phagocytic immune cells in response to the LPS challenge (57).

Emerging evidence from Bisht et al. suggests that microglia activity regulates neurovascular function and structure (58). Specifically, they identified key signaling pathways that recruit microglia to capillaries and precapillary arterioles, estimating that approximately 30% of microglia are capillary-associated microglia (CAMs). In conditions of capillary stalling, enhanced endothelial interactions at the capillary wall are associated with sustained morphological changes in microglia toward activated states, even in the absence of overt blood–brain barrier leakage (51). Consistently, other studies have reported reduced microglial ramification, suggesting microglia activation, in response to localized drops in cerebral blood flow near the microglial soma (59).

Although there is evidence that activated microglia interacts with stalled capillaries (59, 60), the associated augmentation to activated microglia morphology can be a direct result of blood–brain barrier leakage or neuronal death. However, in our study, we did not see significant blood–brain barrier leakage via Evans Blue injections. Limitations of this analysis include the broad age range of mice used in the study. Although both LPS groups contain an equal representation of young and aged mice, the limited sample size constrains the ability to perform a robust age-matched analysis. Moreover, the use of 100% oxygen with an isoflurane anesthetic may not optimally reflect physiological capillary dynamics compared to imaging under medical air or in awake, behaving animals. Future studies should prioritize awake imaging to better capture in vivo microvascular function. Nevertheless, the distinct differences between capillary transit changes in LPS groups compared to SHAM groups indicates the possibility of SCaRe in inferring immune-mediated capillary dynamics.

Our approach remains agnostic to imaging cerebral capillaries, solely dependent on the estimated velocity profile, which may prove useful for assessing function in other organs. However, a limitation of SCaRe is its dependence on accurately tracking a microbubble over seconds, highlighting the importance of preceding algorithms involved in motion correction, clutter filtering, localization, and tracking. All experiments were conducted transcranially, through intact skin and skull, eliminating the need for cranial windows that can alter intracranial pressure and disrupt natural flow dynamics. However, this approach compromises signal-to-noise ratio and distorts the microbubble point spread function, reducing the ability to track microbubbles over time. Therefore, minimizing motion artifacts through proper stereotaxic fixation, applying motion correction, and incorporating aberration correction techniques becomes even more critical. The incorporation of LE-SVD and continuous “gap-less” acquisitions show promise in recovering slower microbubble flows, enhancing our ability to spatiotemporally pair tracks and delineate the complete vascular travel path as well as decrease the scan-time needed to perform ULM. However, the success of SVD-based spatiotemporal filtering depends critically on the input data and how well the singular value spectrum represents separable tissue motion clutter from microbubble signal, where the optimal cutoff is not fixed but varies depending on experimental conditions, such as flow velocity, signal sparsity, and motion levels. Therefore, although here we show how LE-SVD facilitates recovering slower flow microbubbles, increasing the number of retained singular values may degrade, rather than enhance, the separation quality depending on the acquisition of the raw data.

A characteristic feature of the capillary mesh is the existence of thoroughfare channels, allowing RBCs to flow without traversing the density of the capillary mesh during times of low metabolic need and pressure regulation (61). Our data (*SI Appendix*, Fig. S6) seems to indicate that overlapping paths do exist and present closely to expected behaviors observed via optical microscopy. However, the difficulty of tracking 3D structures in a 2D slice presents obstacles in their interpretation. Since our technique was implemented in 2D, we deliberately selected a relatively large elevational slice thickness to maximize the likelihood of capturing complete capillary paths. While this approach improves coverage, it also increases the risk of misidentifying capillary stalls. Thus, a thinner slice thickness would enhance confidence in stall detection at the cost of reduced spatial sampling. Ultimately, 3D ULM represents the ideal configuration for capillary-scale imaging. However, several challenges currently limit the implementation of SCaRe in 3D ULM, including the substantial data requirements needed to maintain equivalent temporal sampling for LE-SVD, and the added complexity of accurately tracking microbubbles in four dimensions (3D + time).

We acknowledge that a barrier to clinical translation of ULM is the long scan times necessary for micron-resolution reconstruction of the microvasculature. However, in the context of capturing rare events such as capillary transit and stalling, we question whether reducing scan time is always desirable. The assessment of capillary function may prove useful for diagnostic or prognostic purposes, thus longer scan durations may, in fact, be necessary to reliably capture these infrequent but physiologically meaningful events and aid clinical assessment of the underlying neuropathology. Given the limited scan-times, exacerbated by the rare occurrence of fully trackable U-shaped microbubble paths inside the field of view, we are only sampling the function of limited number of single capillaries—a subset of the entire capillary mesh. In fact, our sequential Monte Carlo simulations indicate that scan times greater than 10 min are required to populate the whole capillary mesh, given an equal probability of microbubbles flowing through every path, disregarding the existence of thoroughfare channels, the influence of probe characteristics (frequency, elevational slice thickness, transcranial sensitivity), and the influence of hemodynamics under anesthesia. Thus, future optimization is needed to assess the sufficiency of SCaRe within the context of the neurological condition of interest. Nonetheless, SCaRe now can tell us whether a single trajectory is a capillary without the need for multiple tracks for vessel recognition, offering opportunities for noninvasive vessel classification.

## Materials and Methods

### Experimental Design.

The objective of this study was to engineer a methodology that can identify and quantify single capillary function using ULM. To do this, we used in silico hemodynamic mouse brains to investigate the expected behavior of microbubbles freely flowing in the vasculature. Here, we developed a simulation framework that was used to investigate the effects of SVD clutter filtering. Then, the SCaRe methodology was applied and validated in vivo (n = 6). Last, SCaRe was used to identify capillary stalls throughout the whole brain in a neuroinflammation mouse model (n = 4).

### Simulations.

In silico simulations were built on top of a computational model of mouse brain microvasculature with fully connected capillary networks (29). See *SI Appendix*, *Supplementary Methods*. For one hemisphere, there are 291,372 possible paths through capillaries. Thus, each microbubble was forward simulated considering the size of the bubble, the flow velocity (calculated from instantaneous blood flow and vessel diameter), as well as the pulse wave velocity (modulating microbubble positions in accordance with a traveling pulse wave), simulated as a traveling wave of 25 mm/s (62) at 500 BPM (38, 39). Additional constraints were put so that a bubble cannot pass through a capillary smaller than its diameter. Afterward, microbubble datasets were stitched together, synchronous with the cardiac cycle, to produce a fully populated dataset of microbubbles moving through the mouse cerebral microvasculature.

To further simulate ultrasound data from the microbubble dataset, we aligned the microvasculature with an open-source repository for mouse micro–CT (42). Then, a 3D portion of the microbubbles and skull micro-CT were segmented to fit within the elevational and lateral footprint of a 16 MHz 128-element probe (spacing = 100 µm, element height = 1.5 mm). 12 scatterers per resolution cell (63) (24.64 µm × 24.64 µm × 24.64 µm) were placed on a grid of size (12.7 mm in X, 8 mm in Z, and 1.5 mm in Y at a λ/4 spacing) and arranged according to the intensity of the micro-CT so that the scatterers placed at the skull were of higher concentration than the brain matter. These scatterers were superimposed with the centers of the microbubbles for input into a linear ultrasound simulator. A GPU-accelerated simulator based on the equations from SIMUS (64) was used to simulate IQ quadrature demodulation data before beamforming for 3,500 plane waves (7 angles between −11° and 11°) at 7,000 Hz PRF. Simulated results were stored so that the full IQ datasets were linearly superimposed from microbubbles and from skull clutter.

### Animals.

All animal experiments were performed in accordance with the Animal Research Ethics Committee of the Montreal Heart Institute (Protocol # 2023-32-02 TAC-ultrasons). C57BL/6 wild-type mice were employed in this study, (n = 2 male, n = 2 female, 8 to 10 wk old). For LPS experiments n = 12 mice, 6 wk to 8 mo old, were used, n = 6 (n = 3 male, n = 3 female) negative control and n = 6 (n = 3 male, n = 3 female) LPS neuroinflammation challenge. Animals were anesthetized with isoflurane (2%, 1L 100% O_2_ induction; 1 to 1.5%, 0.5 to 1 L O_2_ management). After induction, mice were secured in a stereotaxic frame (SGM-4, Narishige), dehaired at the tops of the heads, and catheterized for tail-vein injections using 27G needles. Physiological saline was used to test vessel patency and confirm tail-vein placement. Degassed ultrasound gel was placed on top of the head, and a warm-water bath was placed immediately on top to couple the transducer to the head.

Microbubble bolus injections were prepared using 4 µL/g of body weight of Definity microbubbles (Lantheus, MA), diluted in physiological saline at a 1:20 ratio. One bolus was used, up to a max of 4, for each ultrasound scan lasting 4 to 6 min. The injection of the bolus was immediately followed by a 50 to 60 µL sterile saline wash. For continuous infusion experiments, a 1:50 dilution was used, and a motorized syringe pump (Harvard Apparatus, Holliston, MA) was used to inject a single bolus over 5 min. Systemic inflammation was triggered by lipopolysaccharide (LPS) injections purified from Escherichia coli strain O111:B4 (Sigma-Aldrich, St. Louis, MO) at a single dose of 2 mg/kg in 200 μL saline. LPS injections were applied intraperitoneal after isoflurane induction and an initial bolus scan for baseline SCaRe mapping. Additional boluses and scanning were performed at 1- and 2-h post LPS injection to assess changes in dwell time, an indicator of capillary transit-times. Four mice (n = 2 LPS, n = 2 SHAM) were injected with Evans blue during tail vein bolus injections to measure blood–brain barrier leakage.

### Immunohistochemistry.

Four mice (n = 2 LPS, n = 2 SHAM) were used for the histological analysis of microglia morphology. Brains were extracted approximately 30 min after the last bolus injection of microbubbles after the 2 h timepoint, postfixed in 4% PFA in phosphate buffer (PB; 0.1 M, pH 7.4), and transferred to a 30% sucrose solution for 48 h for cryoprotection before sectioning. 50 micron sections were used for free-floating immunohistochemistry with overnight incubation in the anti-Iba1 antibody at 4 °C (Wako, 1:1,000), biotinylated anti-rabbit (1:200, Vector Laboratories) was used as a secondary antibody followed by incubation in the avidin–biotin complex for 1 h (Vector Laboratories), finally immunodetection was performed with 3′3-diaminobenzidine (DAB) staining kit (Vector Laboratories) as described previously (65). Whole brain slides were digitized on an automated pathology scanner using a 20x objective (Zeiss, Axioscan 7). Capillary stalling hotspots were first computed and used to segment ROIs from the histological slices and were exported for further analysis in ImageJ.

### Microglia Morphology Analysis.

ROIs were imported to Fiji (66) for analysis with the MicrogliaMorphology macro plugin (54). Briefly, microglia images were first segmented, filtered, and separated into individual cells. Cells were then skeletonized and imported into R for clustering into different morphology categories using fuzzy k-means clustering on the first three principal components. These groups were characterized as amoeboid, ramified, hypertrophic, and rod-like morphologies. Microglia images were overlaid with their clustering, and the proportion of each cluster was analyzed for each ROI.

### Ultrasound Acquisition.

All in vivo ultrasound data were acquired transcranially through intact skin and skull using a linear hockey-stick array with a central frequency of 10.5 MHz and a bandwidth between 5 and 15 MHz (L8-18iD, GE, IL) in LPS animals or a 15 MHz linear array with a bandwidth between 14 and 22 MHz in wild-type animals: both interfaced with a 256 Vantage research ultrasound machine (Verasonics, WA). Central frequency for the 10.5 MHz transducer was set to 10.4167 MHz, and the subsequent sampling rate was 41.7 MHz as determined by the Verasonics Vantage 256. The center frequency was set to 15.625 MHz and a sampling rate of 62.5 MHz for the high frequency 15 MHz array. We emit 4 cycle planewave pulses in transmit and receive using the BS50BW sampling mode in Verasonics (50% bandwidth sampling of the demodulated frequency after digitization of the analog receive signals). Seven tilted plane waves (−5° to −5°) at a compounded frame rate (1,000 Hz for in vivo; 900 to 1,700 Hz for SVD evaluation) for a total of 8,100 to 15,300 Hz PzxRF were acquired for a single dataset. Ultrasonic parameters were chosen to be less than or equal to the transfer time of four contiguous datasets to an NVMe SSD in RAID0 configuration so that data can be continuously acquired and streamed. Thus, for example, if it takes 0.5 s to save four datasets [128 channels × 7,000 frames] determined by the write speed of the SSD, it should take 0.5 s to acquire 7,000 frames (determined by the parameters of the Verasonics Vantage) to ensure gapless transition and ensemble coherence. A single ULM scan was taken between 3 and 5 min, acquiring 350 to 400 GB of data. Offline, all IQ data were beamformed onto a uniform grid with spacing of $\frac{\lambda }{2\surd 2}$ using standard GPU-based delay-and-sum beamforming.

A posterior brain slice, corresponding to Plate 83 of the Allen Brain Atlas, was selected to minimize skull-induced artifacts (at 10 to 15 MHz central frequencies) and to access key brain regions, including the thalamus, hippocampus (CA1 and CA3), and the auditory and visual isocortex. Coregistration was performed by manual matching brain shape and vascular landmarks to the histological slice. This plane lies between the lambda and bregma sutures, which introduce acoustic aberrations and shadowing. By imaging between these sutures, we reduced transmission-related artifacts and improved visualization of deep and superficial brain structures especially in older mice where skull-thickness degrades image quality.

### ULM.

ULM tracks were processed using a sliding window of 6 s with an interval of 1 s (6,000 total frames). Overlapped detections were not taken into consideration at this time. IQ data underwent normalization by first removing the mean in slow time, followed by application of conventional SE-SVD, SE-SVD stitched, or LE-SVD. See *SI Appendix*, *Supplementary Methods* for details. The power frequency spectrum was computed via a periodogram with a 0.2 Tukey apodization sorted by decreasing eigenvalues (24). For each condition, the first 20 eigenvalues were eliminated to remove stationary tissue signal from the skull and brain tissue, leaving only the blood and microbubble signals through empirical removal of significant tissue clutter.

Subsequently, a spatiotemporal tracking algorithm was employed to track microbubbles in space and time (67). This approach facilitated direct access to the spatiotemporal signal and velocity of the track, which was utilized for hidden Markov model (HMM) categorization. The 3D Hessian-based vesselness filter inside the TAL methodology (67) constructs vessels in 3D space (X, Z, and time). To enhance continuity in the slow-time dimension, we adjusted the temporal eigenvalue (ε = 1.5) to better capture temporal coherence in the vessel profiles and adjusted the spatiotemporal tracker to incorporate track pairing. Last, backscattering amplitude (45) was recorded for each track as the absolute value of the IQ and the mean value of all the tracks per pixel were subsequently used for display on ULM and SCaRe maps.

The output of the centerline thinning algorithm may segment multiple tracks from a single microbubble, thus these tracks were first connected before iteratively connecting trajectory beginning and endings spatially within 1λ and temporally within 100 ms. Next, a Kalman filter with RTS smoothing was used before Radial Symmetry superlocalization (*SI Appendix*, *Supplementary Methods*). Finally, these tracks were interpolated and accumulated on a superresolved grid of λ/32, and the velocities were displayed over the track density map.

### SCaRe Processing.

SCaRe processing encompasses three main stages: HMM training, state prediction, and capillary categorization. To recapitulate, continuous data acquisition, individual datasets are beamformed using delay-and-sum (DAS) processing. These datasets are then stitched together post hoc and used as input to the LE-SVD. The resulting microbubble-only datasets preserve slow-flow signals, which are subsequently tracked using the TAL-ULM pipeline (Hessian-based vesselness filtering in both space and time, followed by centerline thinning, Radial Symmetry localization, and Kalman filtering) (59). The resulting tracks can either be accumulated into a density map, as done in standard ULM, or passed through a hidden Markov model for vessel classification. Capillary-classified tracks are then used to compute dwell times, which are aggregated into a SCaRe map. The whole SCaRe pipeline is illustrated in *SI Appendix*, Fig. S5. Initially, all tracks undergo preprocessing to convert velocity profiles into observations comprising four states based on velocity and acceleration. Subsequently, all observations are standardized to the same length to facilitate HMM training using the Baum–Welch algorithm implemented in MATLAB’s hmmtrain function. The initial transition probabilities are represented by a 2 × 2 matrix, while the emission probabilities are structured as a [2 × 4] matrix (68). In the second stage, state predictions on tracks are generated using the Viterbi algorithm (hmmviterbi), estimating whether the profile corresponds to a high-velocity state or a low-velocity state indicative of capillary flow.

Finally, in the third stage, these states are characterized based on the occurrence and location of low-velocity states. Specifically, we identify whether the capillary network exhibits a U-shaped profile with a minimum capillary transit time threshold of 0.120 s. Here, Capillary Transit Time is defined as the duration spent in the low-velocity state between the inlet and outlet points. For visualization purposes, capillary trajectories are processed by accumulating centered positions within the HMM Viterbi segmented regions of the superlocalized track positions. The subsequent map thus shows capillary dwell time, an indirect measurement of transit-time, as the integral over time given by the frame rate, highlighting pixels where microbubbles remain stationary, indicating time spent in the capillary given by

$$I_{\mathrm{Dwell}\;\mathrm{Time}}(x,z)=\int_{t1}^{t2}\sum_{i=1}^{N}\delta \left(x-x_{i}\left(t\right),z-z_{i}\left(t\right)\right)dt,$$

where t1 and t2 indicate the beginning and end of the ultrasound scan, N indicates the number of capillary tracks, and δ is a Dirac delta function placed at the capillary track coordinates summated over time. Subsequently, SCaRe maps are superimposed onto gray-scale ULM maps of all trajectories, effectively integrating functional and structural information for comprehensive visualization and analysis.

To quantify capillary stalling, HMMs were used to classify possible tracks that enter or exit a capillary vessel. Each entrance track was paired across the scan time with an exit track only if they occur within 10 µm of each other (Euclidean distance). The locations, the travel path, and the stalled times were recorded for each pair and a Gaussian convolved map was overlaid onto ULM images to create stalling hotspot maps for ROI segmentation in histology images. A flow map of the entire SCaRe pipeline from acquisition to capillary mapping is shown in *SI Appendix*, Fig. S5.

Baseline capillary dwell time measurements were taken for a C57BL6 wild-type mouse. The Allen Brain Atlas plate 68 was used to parcellate brain regions and mean and max dwell time were recorded for each region. Specific regions such as the Striatum, Corpus Callosum, Hippocampus, Thalamus, and the Barrel Field Cortex were segmented, and distributions were compared. Additionally, preferential capillary pathways were identified by calculating similarity between SCaRe trajectories using dynamic time warping and matching trajectories were overlaid onto a λ/30 (~3.2 µm) resolution map (*SI Appendix*, Fig. S6).

### Statistical Testing.

A G*Power a priori analysis was performed to estimate the required sample size for a two-way repeated-measures ANOVA. Assuming an effect size of 0.5, two groups, and a correlation of 0.9 among repeated measures, we calculate for a feasibility study of six animals per group. Distributions of capillary dwell time in negative control (n = 6) and LPS-injected mice (n = 6) for the entire 4- to 6-min scan (at three time points: 0 h-baseline, 1 h-post, and 2 h-post) were quantitatively compared. We used two-way ANOVA for LPS analysis and Type II Wald chi-squares test for microglia morphological analysis (see *SI Appendix* for details).

## Supplementary Material

Appendix 01 (PDF)

Movie S1. Sequential Monte Carlo simulations of free-flowing microbubbles through a microvascular mouse brain network with fully connected capillaries.

Movie S2. SIMUS simulated microbubbles from Sequential Monte Carlo simulations overlaid with skull scatterers before and after SVD filtering.

Movie S3. Effect of ensemble size on recovering stationary microbubbles that pass through capillaries.

Movie S4. SCaRe ULM of a single microbubble passing through a capillary *in vivo*.

Movie S5. Dynamic SCaRe ULM all single capillary tracks temporally aligned perfusion.

Movie S6. Effects of short and long ensemble SVD in a single microbubble track inLPS injected animals.

## Data Availability

Processing codes for single capillary reporters are located in a repository found at https://github.com/ProvostUltrasoundLab/SingleCapillaryReporters (69) where an example script is provided with the corresponding datasets (https://doi.org/10.20383/103.01507) (70). The data repository also contains datasets of the simulated microbubble results for a high frequency ultrasound probe with ground truth microbubble position data flowing through a 2D slice of the mouse brain cortex. All other data are included in the manuscript and/or supporting information.
