# Supplementary material for: Assessing cerebral capillary function and stalling using single capillary reporters in ultrasound localization microscopy
Source: Proc Natl Acad Sci U S A. 2026 Jan 9;123(2):e2509564123. doi: 10.1073/pnas.2509564123 (PMC12799139; doi:10.1073/pnas.2509564123)
Supplement: Supplementary file 1 — Appendix 01 (PDF) [file pnas.2509564123.sapp.pdf]

PNAS

Supporting Information for
Assessing Cerebral Capillary Function and Stalling using Single Capillary Reporters in Ultrasound Localization Microscopy

Stephen A. Lee[1]*, Alexis Leconte[1], Alice Wu[1], Joshua Kinugasa[2], Gerardo Ramos Palacios[3,4], Jonathan Porée[1], Abbas F. Sadikot[3,4,5], Andreas Linninger[6], Jean Provost[1,7]*

[1]Department of Engineering Physics, Polytechnique Montreal; Montreal, H3T 1J4, Canada.
[2]Department of Biomedical Engineering, Chiba University; 263-8522, Japan.
[3]Department of Neurology and Neurosurgery, McGill University; Montreal, H3A 2B4, Canada.
[4]Montreal Neurological Institute and Hospital
[5]William Cone Laboratory for Neurosurgery Research
[6]Department of Biomedical Engineering, University of Illinois Chicago, 60607, USA.
[7]Montreal Heart Institute; H1T 1C8, Canada.

Corresponding: *Stephen A. Lee, *Jean Provost
Email: Stephen.lee@polymtl.ca, jean.provost@polymtl.ca

This PDF file includes:

Supporting text
Figures S1 to S6
Tables S1 to S5
Legends for Movies S1 to S6

Other supporting materials for this manuscript include the following:

Movies S1 to S6

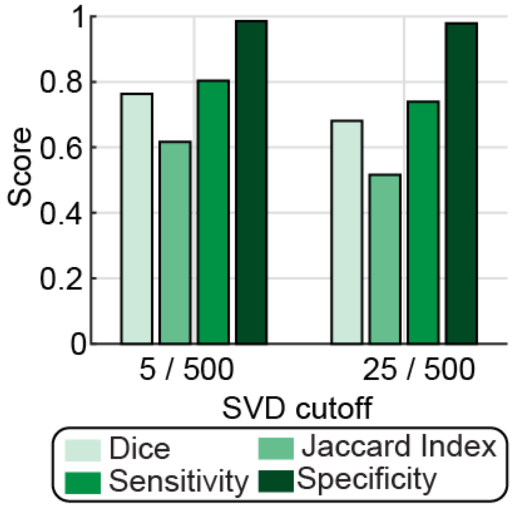

Fig S1. Effect of SVD threshold on *in silico* capillary reconstruction. Comparisons were made between clean, no-clutter MB ULM reconstructions and SVD filtered ULM reconstructions, removing 5 and 25 eigenvalues out of an ensemble size of 500. Specificity remains high while small decreases are seen in sensitivity. Larger changes are seen in Dice score and Jaccard Index indicating loss of precision while maintaining ability to reconstruct larger vessels.

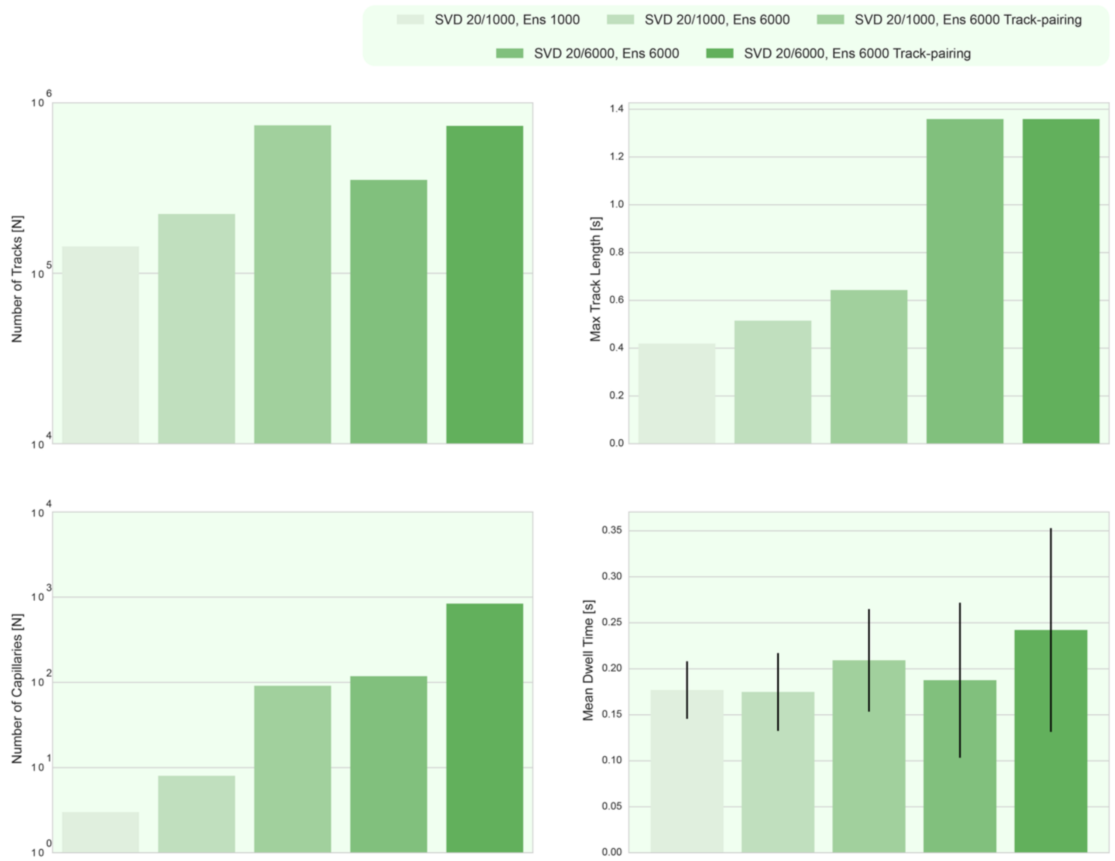

Fig. S2. Effect of ensemble size & track pairing on track lengths and number of recoverable capillaries in wild-type mice. SVD x/y indicates x removed from y singular values. Ens indicates the ensemble size or number of frames used for tracking. Number of tracks indicates how many total tracks were collected after a whole 5-minute scan. Max Track Length indicates the longest tracks. Number of capillaries indicates the number of tracks that are state predicted by the hidden Markov model to be capillary microbubbles. Mean dwell time shows mean value of capillary transit time per condition.

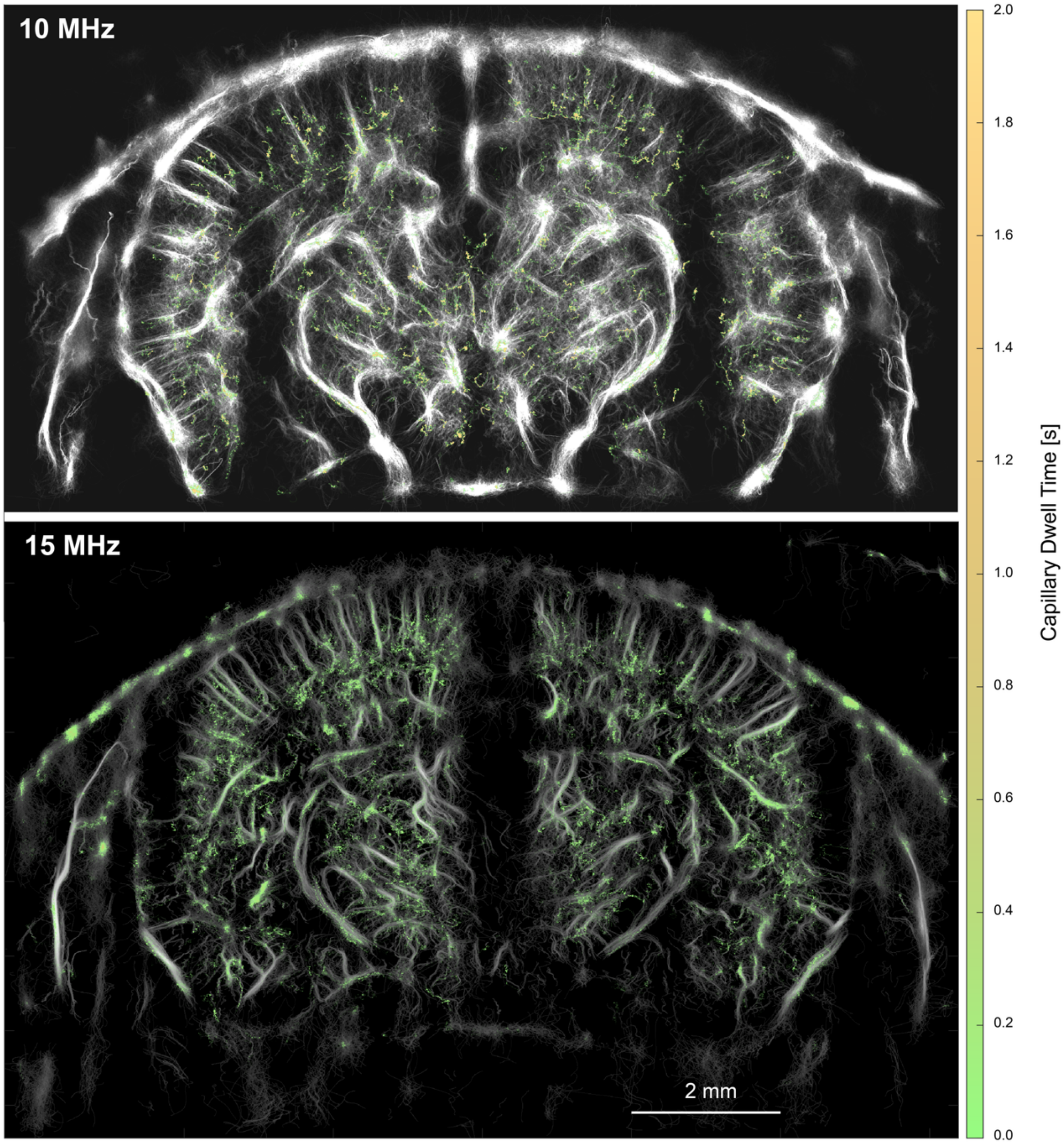

Fig S3. SCaRe map comparison between a 10.4 MHz and 15.6 MHz linear array. Both images were constructed on a ULM acquisition using continuous perfusion of MBs for 5 minutes in separate wild-type mice.

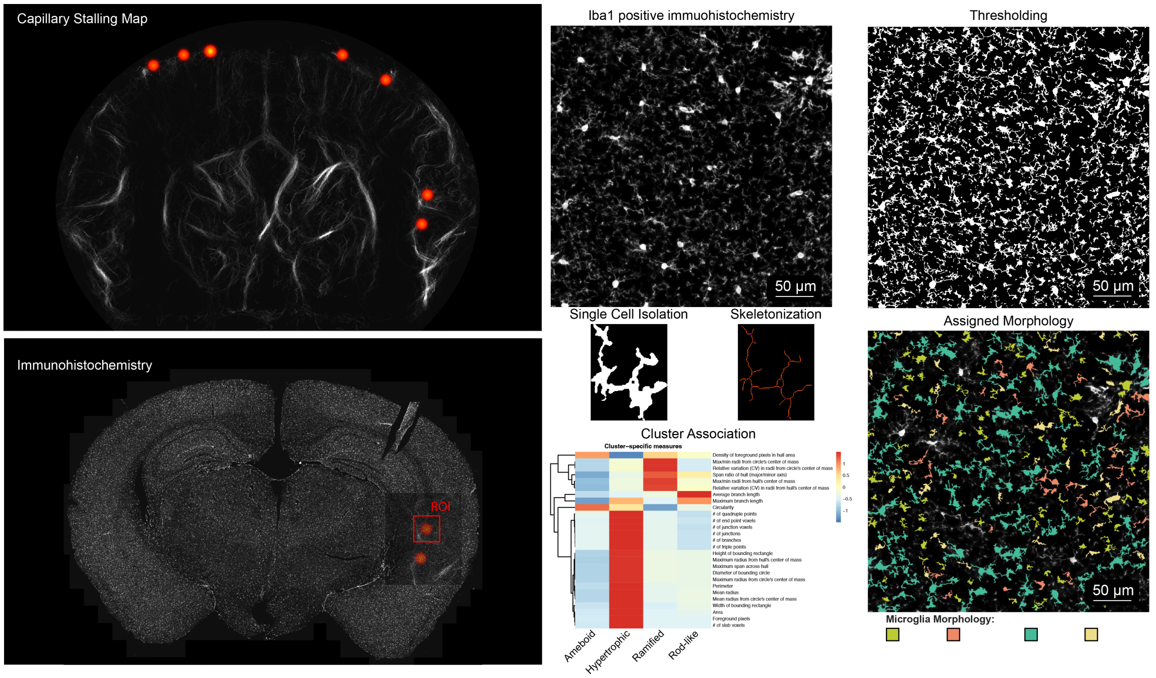

Fig. S4. Capillary Stalling ROI selection on Immunohistochemistry slices. For each ROI, Iba1 positive microglia are thresholded and skeletonization is performed on single cells for cluster analysis and remapping onto histological slices.

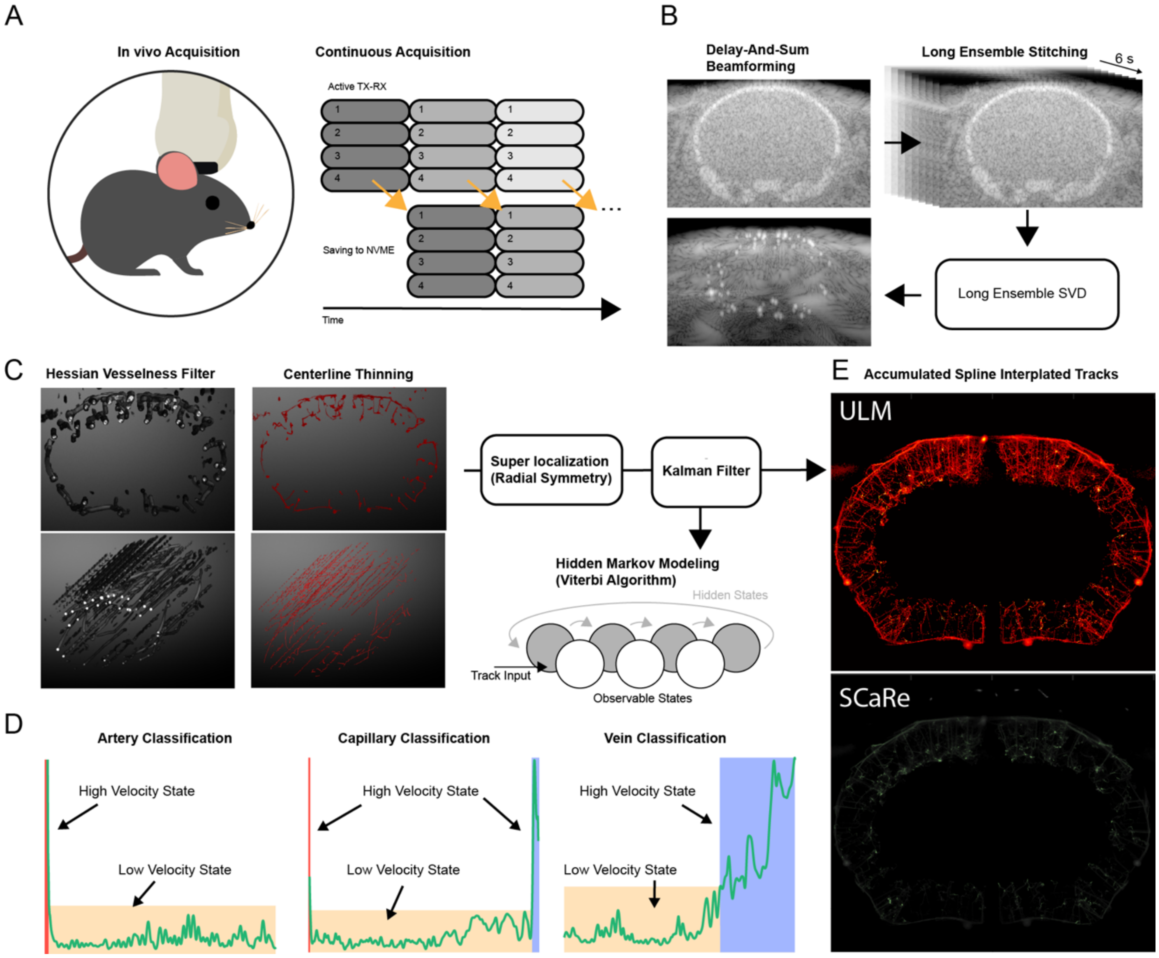

Fig. S5. Flow diagram of the entire SCaRe Pipieline. A) The data pipeline starts from an in vivo acquisition through intact skin and skull of the mouse brain. The acquisition consists of a gap-less and circular scheme where the transmit and receive events were timed precisely to the saving time on the disk. **B)** After dataset acquisition, individual datasets were beamformed using delay and sum beamforming and stitched together for long ensemble SVD clutter filter processing. The result are isolated microbubbles with improved sensitivity to slow flow. **C)** Long-ensemble isolated microbubble data are then passed through a hessian-based vesselness filter to create 3D tracks over space and time of the size of the point-spread-function. These tubes are then centerline thinned to gain an *a priori* estimation of the microbubble center. These centers were updated through Radial Symmetry localization as well as forward and backwards Kalman filtering. Here the results are super-resolved tracks for all microbubbles within an ensemble and the tracks can be accumulated onto a density map or input for SCaRe capillary classification. **D)** Microbubble tracks were estimated for high and low states via the Viterbi Algorithm for hidden Markov modeling. Thus, tracks that correspond to arteries (high - low), capillaries (high - low - high), and veins (low - high) can be found within all possible tracks. E) Resultant ULM and SCaRe maps from the tracks after tracking and localization. Here ULM is shown as the density (number of total tracks within a pixel) and SCaRe is shown as a function of dwell time (cumulative time a microbubble spends inside a pixel).

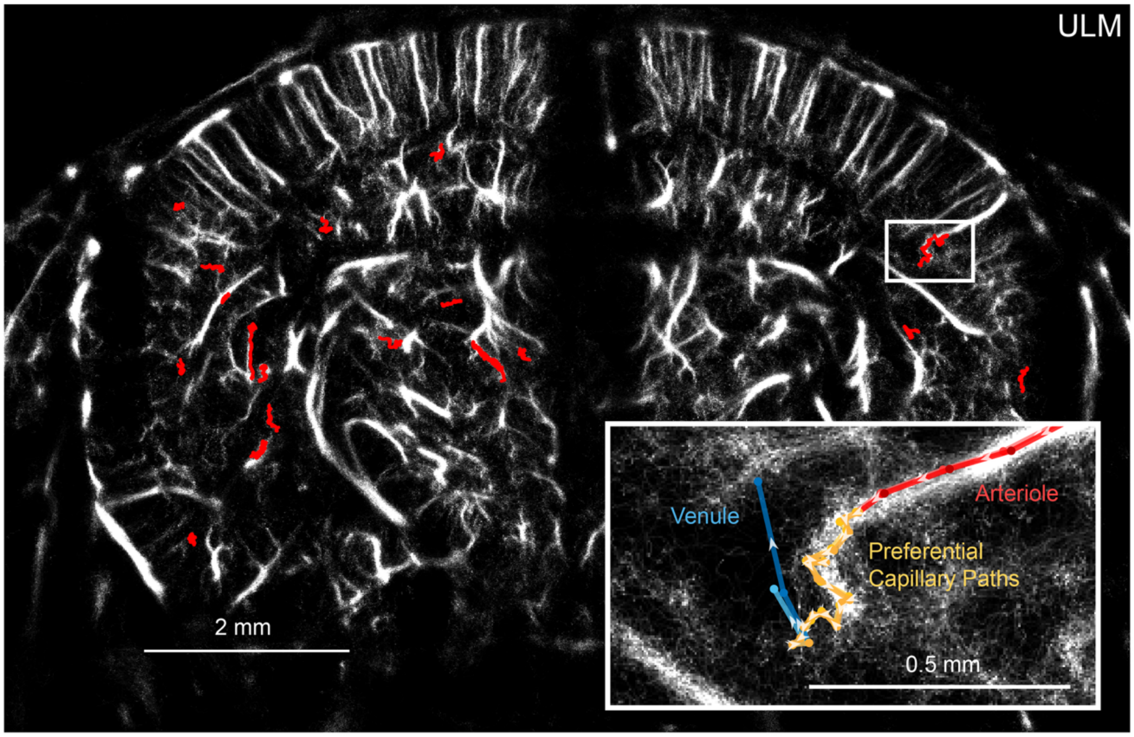

Fig. S6. Identification of preferential capillary pathways using distance minimization. Capillary trajectory path similarity estimated through dynamic time warping. Trajectories with errors less than 1 micron were overlaid onto ULM maps. Subfigure demonstrates two trajectories that follow the same capillary pathway indicating preferential channels from arteriole to venule.

Table S1. Benchtop for Localization and Tracking (BLT) simulation parameters for flowing microbubbles through a capillary-connected microvascular network

Microbubble Simulation Parameters

Main parameters				
dt	:	0.001	|	[s] simulation sample step size
total_simulation_time	:	300s	|	[s] max bubble track length time
graph_layout	:	"capillary"	|	for fully connected capillary graph network
bubbles parameters				
nBubbles	:	50,000	|	number of unique bubbles in dataset
size_mu	:	2	|	[μm] mean value of microbubble size distribution
size_sigma	:	3	|	[μm] standard deviation of microbubble size distribution
pulse parameters				
PWV	:	25	|	[mm/s] injected pulse wave velocity
BPM	:	300	|	cardiac heart rate beats per minute
min_poiseuille	:	0.3	|	minimum poiseuille flow factor to prevent boundary stalling
nSamples_poiseuille	:	1000	|	n samples used to calculate poiseuille flow distribution
min_CBF	:	0.003	|	mininum flow value to prevent nodal stalling
sim parameters				
min_length	:	20	|	minimum length of microbubble tracks
framing parameters				
nBubbles	:	3000	|	n bubble to generating a a fully populated dataset
total_simulation_time	:	60	|	total time for fully populated dataset lines
MBsize_factor	:	1	|	display only factor for artificially increasing the display size

Table S2. Two-Way ANOVA testing of capillary frequency over time. Group indicate LPS or SHAM, Time indicates timepoints. Group*Time indicates testing for interaction.

Source	Sum Sq.	d.f.	Mean Sq.	F	Prob > F	Significant?
**Group**	0.47095	1	0.47095	3.92	0.0569	n.s.
**Time**	0.23171	2	0.11585	0.97	0.3924	n.s.
**Group*Time**	1.08822	2	0.54411	4.53	0.019	*
**Error**	3.60101	30	0.12003			
**Total**	5.39188	35				

Table S3. Two-way ANOVA multiple comparisons for capillary stalling frequency over time.

Comparison	Lower Limit	Difference	Upper Limit	P-value	Significant?
**LPS_base x SHAM_base**	-0.4304	0.1780	0.7864	0.9460	n.s.
**LPS_base x LPS_1h**	-1.0052	-0.3968	0.2116	0.3746	n.s.
**LPS_base x SHAM_1h**	-0.3275	0.2809	0.8893	0.7242	n.s.
**LPS_base x LPS_2h**	-0.3952	0.2132	0.8216	0.8908	n.s.
**LPS_base x SHAM_2h**	-0.5646	-0.5646	-0.5646	-0.5646	n.s.
**SHAM_base x LPS_1h**	-1.1832	-0.5748	0.0336	0.0726	n.s.
**SHAM_base x SHAM_1h**	-0.5055	0.1029	0.7113	0.9952	n.s.
**SHAM_base x LPS_2h**	-0.5732	0.0353	0.6437	1.0000	n.s.
**SHAM_base x SHAM_2h**	-0.7426	-0.1342	0.4742	0.9838	n.s.
**LPS_1h x SHAM_1h**	0.0693	0.6777	1.2861	0.0221	*
**LPS_1h x LPS_2h**	0.0017	0.6101	1.2185	0.0491	*
**LPS_1h x SHAM_2h**	-0.1678	0.4406	1.0490	0.2662	n.s.
**SHAM_1h x LPS_2h**	-0.6761	-0.0677	0.5407	0.9994	n.s.
**SHAM_1h x SHAM_2h**	-0.8455	-0.2371	0.3713	0.8402	n.s.
**LPS_2h x SHAM_2h**	-0.7779	-0.1695	0.4389	0.9559	n.s.

Table S4. Test for independence between clusters (microglia morphology proportions) and treatment (LPS/SHAM). Analysis of Deviance Table (Type II Wald chi square tests). (*) $p < 0.05$, (**) $p < 0.01$, (***) $p < 0.001$.

	**Chi square**	**Df**	**Pr(>Chisq)**	**Significant?**
**Cluster**	37.2596	3	4.257e-08	***
**Treatment**	0.1095	1	0.7408	n.s.
**Cluster:Treatment**	30.2985	3	1.194e-06	***

Table S5. Post-hoc significance testing with Bonferroni correction for microglia morphology. Results are given on the log odds ratio (not the response) scale.

**contrast**	**Cluster**	**estimate**	**SE**	**df**	**z.ratio**	**p.value**	**Significant?**
PBS - LPS	Ameboid	0.1880784	0.1454600	Inf	1.293	0.7841	ns
PBS - LPS	Hypertrophic	-0.6251256	0.1772597	Inf	-3.527	0.0017	**
PBS - LPS	Ramified	0.6077160	0.1596792	Inf	3.806	0.0006	***
PBS - LPS	Rod-like	-0.2116194	0.1563722	Inf	-1.353	0.7038	ns

Movie S1 (separate file). Sequential Monte Carlo simulations of free-flowing microbubbles through a microvascular mouse brain network with fully connected capillaries.

Movie S2 (separate file). SIMUS simulated microbubbles from Sequential Monte Carlo simulations overlaid with skull scatterers before and after SVD filtering.

Movie S3 (separate file). Effect of ensemble size on recovering stationary microbubbles that pass-through capillaries.

Movie S4 (separate file). SCaRe ULM of a single microbubble passing through a capillary *in vivo*.

Movie S5 (separate file). Dynamic SCaRe ULM all single capillary tracks temporally aligned perfusion.

Movie S6 (separate file). Effects of short and long ensemble SVD in a single microbubble track in LPS injected animals.
